# PRECEPT-OHCA: prehospital evaluation of confidence and practice in the treatment of out-of-hospital cardiac arrest across Australia, the United Kingdom, and New Zealand: a cross-sectional study protocol

**DOI:** 10.1016/j.resplu.2026.101444

**Published:** 2026-08-08

**Authors:** Salman Naeem, Stewart Ashcroft-Quinn, Alison Brown, Amy Keir, Nathan West, Charmaine Gray, Lisa Ramage, Danyal Bakht, Leah Armit, Tim Harris, Shahid Ullah, Michael D. Christian

**Affiliations:** a Department of Emergency Medicine, Flinders Medical Centre, Southern Adelaide Local Health Network, Adelaide, South Australia, Australia; b College of Medicine and Public Health, Flinders University, Adelaide, South Australia, Australia; c Department of Emergency Medicine, East Kent Hospitals University NHS Foundation Trust, Kent, England, the United Kingdom of Great Britain and Northern Ireland; d Clinical Services Directorate, South Australia Ambulance Service, Adelaide, South Australia, Australia; e Department of Pre-Hospital Care, MedSTAR Emergency and Retrieval Service, South Australia, Australia; f Department of Emergency Medicine, Barts Health NHS Trust, London, England, the United Kingdom of Great Britain and Northern Ireland; g Consultant Prehospital Emergency Medicine, Essex and Herts Air Ambulance Trust, Colchester, the United Kingdom of Great Britain and Northern Ireland; h Department of Medicine, King Edward Medical University, Mayo Hospital, Lahore, Punjab, Pakistan; i Department of Emergency Medicine, Central Adelaide Local Health Network, Adelaide, South Australia, Australia; j Associate Professor, Biostatistics, School of Medicine and Public Health, Flinders University, Adelaide, South Australia, Australia; k Department of Critical Care Medicine, Aviation/Military Medicine, Pre-hospital Medicine, University of British Columbia, Vancouver, British Columbia, Canada

**Keywords:** OHCA, Cardiac arrest, Out-of-hospital cardiac arrest, Treatment confidence, Survey, Emergency medical services

## Abstract

**Background:**

Out-of-hospital cardiac arrest (OHCA) has poor survival. Resuscitation guidelines recommend consideration of reversible causes, commonly termed the “4 Hs and 4 Ts”, but prehospital constraints may limit clinicians’ ability to identify or exclude them. This study will assess clinicians’ confidence in identifying reversible causes, team composition, and perceived barriers to improving OHCA outcomes.

**Methods:**

This international, multicentre, cross-sectional survey will recruit prehospital clinicians across Australia, New Zealand and the United Kingdom. It will be disseminated through prehospital and emergency medicine organisations using email links and QR codes. The questionnaire assesses respondent characteristics, team composition, equipment and medication availability, and confidence in ruling in and ruling out each reversible cause using a five-point Likert scale. Structured and free-text items examine service practices and barriers. From an estimated population of 54,874 clinicians, at least 382 responses are required. The primary outcome is clinicians’ perceived confidence in identifying reversible causes. Secondary outcomes include service-level standard operating procedures, use of clinical adjuncts, and perceived barriers. Analyses will include descriptive statistics, Mann–Whitney U, Kruskal–Wallis and chi-squared tests, with thematic content analysis of free-text responses.

**Discussion:**

The study will describe international variation in prehospital OHCA practice and assess how adjuncts, including point-of-care ultrasound, may support identification of reversible causes. Self-reported confidence may not reflect diagnostic accuracy or clinical performance.

**Ethics and dissemination:**

Ethics approval was granted by the Southern Adelaide Local Health Network Human Research Ethics Committee [2025/HRE00367] and the UK Health Research Authority [IRAS: 346781]. Findings will be published and presented at conferences.

## Highlights

• International survey of clinician confidence in ruling in or out OHCA reversible causes.
• Compares team composition and diagnostic adjunct availability.
• Identifies barriers to improving prehospital OHCA outcomes.
• Includes services across Australia, New Zealand and the UK.

## Introduction

Cardiac arrest is defined as the loss of mechanical activity of the heart, confirmed by the absence of signs of circulation.^1^ Out-of-hospital cardiac arrest (OHCA) is defined as cardiac arrest occurring outside of a healthcare facility.^2^ It has an annual incidence of 65.4–108.9 per 100,000 person-years globally,^1^, ^2^, ^3^ and 108.9 per 100,000 person-years in Australia, according to the Australasian Resuscitation Outcomes Consortium (Aus-ROC) registry.^3^ This registry compiles data from all regional emergency medical services (EMS) across Australia and New Zealand. Recent statistics underscore the significant morbidity and mortality associated with OHCA, with fewer than 1 in 8 patients (13%) surviving to hospital discharge.^3^ This pattern is echoed globally.^4^ Despite advancements in prehospital care, survival rates have not significantly improved over the past three decades.^5^

The resuscitation councils in the United Kingdom (UK), Europe and Australasia recommend consideration of eight reversible causes of cardiac arrest, commonly referred to as the “4 Hs and 4 Ts,” i.e., hypoxia, hypothermia, hypo/hyperkalemia, hypovolemia, tamponade, tension pneumothorax, thrombosis, and toxins.^6^, ^7^, ^8^ These recommendations are based largely on expert consensus, mostly drawn from in-hospital cardiac arrest (IHCA) data.^9^ There is a lack of robust literature on the causes of OHCA. Much of the existing data stems from patients who survive to hospital admission,^10^ introducing survivor bias. Furthermore, registry data regarding arrest aetiology are often incomplete or inconsistently reported.^11^ In the resource-limited prehospital setting, it may not be feasible to reliably identify or exclude these causes, potentially impacting OHCA outcomes compared to IHCA.

In recent decades, critical care capabilities have expanded in the prehospital setting worldwide.^12^ This includes the use of mechanical cardiopulmonary resuscitation (CPR) devices,^13^ point-of-care (PoC) diagnostics,^14^ and extracorporeal CPR (eCPR).^15^ Across Australia, New Zealand the UK some prehospital services have incorporated advanced diagnostics and highly trained personnel into OHCA management, potentially contributing to regional differences in outcomes.^16^

Identifying the underlying cause of OHCA is crucial. While literature suggests that approximately 60–70% of OHCAs are presumed to be of cardiac origin,^2^ there is limited data detailing the remaining 30–40% of cases. Timely identification and treatment of the underlying cause is critical to achieving meaningful outcomes and may be a key factor in the lack of progress in OHCA survival rates.^17^

Evaluating prehospital clinicians’ confidence in identifying the underlying cause of OHCA can give us insights about the gaps in the application of the advanced life support algorithm. This cross-sectional survey aims to describe the current landscape of OHCA management across EMS in Australia, the UK and New Zealand. It will examine team composition, clinician experience, use of critical care resources and diagnostic adjuncts, perceived clinician confidence in identifying arrest aetiology, and perceived barriers to improving OHCA outcomes.

## Methods

### Research questions

The PRECEPT-OHCA study will address three principal research questions:

1. What is the current composition of prehospital teams responding to OHCA across ambulance and retrieval services in Australia, the UK and New Zealand, including the use of clinical adjuncts and service-level standard operating procedures?
2. What are prehospital clinicians’ perceived confidence in ruling in or ruling out each of the reversible causes of OHCA, commonly referred to as the “4Hs and 4Ts” (hypoxia, hypovolaemia, hypo/hyperkalaemia, hypothermia, tension pneumothorax, tamponade, thrombosis and toxins)?
3. What are prehospital clinicians' perceived barriers to improving outcomes in OHCA management?

### Study design

PRECEPT-OHCA is an international, multicentre, cross-sectional survey study of prehospital clinicians, defined as health care providers working outside the boundaries of healthcare facilities delivering medical care to patients.^18^ The survey will be distributed to prehospital clinicians across ambulance, air ambulance, and volunteer medical services in Australia, the United Kingdom, and New Zealand. Coordination across sites in Australia and New Zealand will be facilitated through the Australasian College of Paramedicine (ACP) and the Australasian College for Emergency Medicine (ACEM), while sites in the United Kingdom will be engaged through the National HEMS Research and Audit Forum (NHRAF) and the National Ambulance Research Steering Group UK (NARSG UK). Each participating organisation will appoint a site champion responsible for local dissemination. The survey will be distributed via email links and QR codes embedded in departmental posters displayed at service locations and will additionally be advertised through social media channels. The survey will be open to responses in all jurisdictions between 1 July 2026 and 31 August 2026. Project data will be collected and managed using REDCap (Research Electronic Data Capture), a secure, web-based electronic data capture platform.^19^, ^20^

### Eligibility criteria

**Inclusion criteria**

• All prehospital clinicians physically involved in the management of out-of-hospital cardiac arrest.

**Exclusion criteria**

• Prehospital clinicians not physically involved in the management of OHCA.

Participants will self-identify against these criteria after reviewing the eligibility information provided at the beginning of the survey.

### Data collection tools and techniques

A structured, anonymised, self-administered electronic questionnaire will be used to collect data. The questionnaire was developed by the core research team to address the study's primary and secondary outcomes, using the “4Hs and 4Ts” framework of reversible causes of cardiac arrest as the structural basis for the confidence-related items. Item generation was informed by review of existing literature, supplemented by the research team's clinical and methodological expertise in prehospital and emergency care. Draft items were iteratively reviewed and refined by the co-investigator group. The survey will be hosted on the REDCap platform using a secure account accessible only to the named study team, with the REDCap subscription provided by Flinders University.^19^, ^20^ Participants will access the survey through a hyperlink or QR code, and it will remain open for two months from the date of distribution. The survey instrument will incorporate five-point Likert-scale questions to assess clinician confidence in ruling in and ruling out each of the reversible causes of OHCA. Multiple-choice questions will explore team composition, use of adjuncts, and service-level practices, while free-text items will capture perceived barriers to improving OHCA management. To mitigate sampling and under coverage bias, clinicians from all participating prehospital organisations across Australia, the United Kingdom, and New Zealand will be invited to participate. Distribution will occur through multiple official communication channels, including ACP, ACEM, NHRAF, and NARSG UK. The survey has undergone pilot assessment by the core research team prior to full distribution to ensure clarity, face validity, and neutrality of question wording. The REDCap platform will be configured such that responses cannot be linked to individual participants, ensuring full anonymity.

Given the fully anonymised nature of the study, no formal written consent process is required. Participant information, including eligibility criteria and consent details, will be provided in digital format within REDCap. Participation is entirely voluntary, and completion and submission of the survey will be deemed implied consent. It will not be possible to withdraw consent due to anonymity of the data submitted. In addition to the survey, prehospital services across the three countries will be invited to share their OHCA management protocols, allowing service-level practices to be contextualised against clinician-reported responses. Data will be exported and checked for completeness, consistency, and plausibility before being amalgamated and analysed using IBM SPSS Statistics version 27 or above. Full details of the data variables collected are provided in Supplementary File 1.

### Sample size

The estimated total survey population across the three countries is 54,874 individuals, comprising approximately 26,071 ambulance service staff in the UK,^21^, ^22^ 26,603 paramedics in Australia,^23^ and 2,200 paramedics in New Zealand.^24^ As no previous large international survey has quantified clinicians' confidence in this context, a conservative prevalence estimate of 50% will be assumed, as this produces the maximum sample size requirement. A sample size calculation for a finite population of 54,874 eligible prehospital clinicians across Australia, New Zealand, and the United Kingdom will be performed using a z-score of 1.96, a proportion of *p* = 0.5, and a margin of error of 5%. The initial uncorrected sample size will be *n*_0_ = 384; applying the finite population correction formula yields a minimum required sample of 382 completed responses. This sample size will allow estimation of the proportion of clinicians reporting confidence with acceptable precision. Composite confidence scores derived from the Likert-scale items will also be calculated and analysed; however, no separate sample size calculation will be performed for these scores, as there is currently insufficient evidence regarding their expected distribution, variability, or clinically meaningful differences. Consequently, analyses of the composite confidence scores will be considered supportive of the primary outcome and interpreted alongside the primary analysis rather than serving as the basis for sample size determination.

### Outcome measures

**Primary outcome**

The primary outcome is the perceived confidence of prehospital clinicians in ruling in and ruling out each of the reversible causes of out-of-hospital cardiac arrest, specifically the 4Hs (hypoxia, hypovolaemia, hypo/hyperkalaemia and hypothermia) and 4Ts (tension pneumothorax, tamponade, thrombosis and toxins), as measured using a five-point Likert scale.

**Secondary outcomes**

Secondary outcomes include: (i) description of current standard operating procedures for OHCA management across participating services; (ii) prevalence and patterns of use of clinical adjuncts, including point-of-care ultrasound (PoCUS), point-of-care (PoC) blood investigations and mechanical cardiopulmonary resuscitation devices; and (iii) prehospital clinicians' perceived barriers to improving outcomes in OHCA management.

### Data analysis

Analyses will be conducted using Microsoft Excel and IBM SPSS Statistics version 27 or later. Statistical tests will be two-sided, with *p* < 0.05 considered statistically significant; however, interpretation will prioritise effect sizes, confidence intervals, clinical relevance, and consistency across analyses rather than p-values alone. The distribution of continuous variables will be assessed using graphical methods, including histograms and Q–Q plots, as well as appropriate statistical tests prior to analysis. Normally distributed variables will be summarised using means and standard deviations (SD), whereas non-normally distributed and ordinal variables will be summarised using medians and interquartile ranges (IQR). Categorical variables will be presented as frequencies and percentages.

Confidence will be analysed for each individual reversible cause using both composite confidence scores and a dichotomised confidence outcome. Composite rule-in and rule-out confidence scores will be derived from the corresponding Likert scale items, with higher scores indicating greater overall confidence. For the dichotomised analysis, confidence will be classified as high confidence (“somewhat confident” or “very confident”) and lower confidence (“unsure”, “less confident”, or “not confident at all”). Individual confidence ratings for each of the 4Hs and 4Ts will also be summarised and reported descriptively.

Confidence outcomes will be compared across prespecified groups, including years of prehospital experience, professional group, service type, frequency of OHCA exposure, organisational training frequency, country, and availability or use of PoCUS. Composite confidence scores will be summarised using appropriate measures of central tendency and dispersion depending on their distribution, and internal consistency will be considered acceptable if Cronbach's alpha ≥0.70. For comparisons involving continuous variables between two groups, Student's *t*-test will be used for normally distributed data and the Mann–Whitney *U* test for non-normally distributed data. Comparisons across more than two groups will use the Kruskal–Wallis test, with post-hoc pairwise comparisons adjusted for multiple testing where appropriate. Dichotomised confidence outcomes will be compared using Pearson's chi-squared test or Fisher's exact test as appropriate. Results will be reported with effect estimates, 95% confidence intervals, and p-values.

Regression modelling will be used to examine factors independently associated with clinician confidence. Composite confidence scores will be analysed using multivariable linear regression if model assumptions are adequately met, with model fit assessed using residual diagnostics and adjusted *R*^2^. Dichotomised confidence outcomes will be analysed using mixed effect regression modelling and reported as odds ratios with 95% confidence intervals; goodness of fit will be assessed using the Hosmer–Lemeshow test and discrimination using the area under the receiver operating characteristic curve (AUC). Candidate explanatory variables will include years of prehospital experience, professional group, service type, frequency of OHCA exposure, organisational training frequency, country, and availability or use of PoCUS. Results will be interpreted with emphasis on effect estimates, 95% confidence intervals, and clinical relevance.

A prespecified subgroup analysis will compare confidence outcomes between clinicians with and without access to PoCUS, with adjustment for experience, professional group, service type, training frequency, OHCA exposure frequency, and country of practice. It will analyse the confidence in ruling in and ruling out cardiac tamponade, hypovolaemia, tension pneumothorax, and thrombosis, as these conditions may be directly informed with PoCUS. Differences between groups will be assessed using the statistical methods described above.

Free-text responses will undergo inductive thematic analysis. AI-assisted coding tools may be used to facilitate data organisation, but themes will be reviewed and refined by the research team. Qualitative themes will be triangulated with quantitative findings to provide a comprehensive account of clinician perspectives and a richer interpretation of practice variation and perceived barriers.

The extent and pattern of missing data will be described for each variable. Complete-case analysis will be used for the primary analyses. Where missing data are substantial or appear systematically related to key participant or service characteristics, sensitivity analyses will be undertaken to assess the potential impact of missingness on the study findings. Where appropriate, multiple imputation may be used as a sensitivity analysis to evaluate the robustness of results to missing data assumptions. The study will be published in accordance with the checklist of reporting od survey studies (CROSS) guidance.^25^ The full protocol of the study is provided in Supplementary File 2.

## Discussion

This study will be the first international study investigating prehospital clinicians’ confidence in ruling in or out the reversible causes of OHCA using the current recommendations by national resuscitation guidelines.^6^, ^7^, ^8^ This will give insight into the application of the advanced life support (ALS) algorithm in resource limited prehospital environment. This may highlight challenges in applying the current ALS algorithm and its recommendations across different cadres of prehospital clinicians in different countries. Confidence is a self-reported construct and may not necessarily reflect diagnostic accuracy or actual clinical performance. However, it is not feasible to assess competence on a large scale using a cross-sectional survey. The authors recognise the limitations of the methodology used.

There have been advancements in prehospital care in the last few decades.^12^ Prehospital critical care resources have been developed along with provision of mechanical CPR devices, point-of care diagnostics and provision of medicines.^12^, ^13^, ^14^, ^15^ The survey aims to access team composition and availability of equipment and medicines. However, access to advanced measures does not necessarily translate into their routine use in OHCA. This limitation has somewhat been addressed by provision of free text box for participants to describe when and why they deviate from the ALS protocols.

Survey distribution and population representation present a viable challenge for any cross-sectional study.^26^ The authors have ensured a multilayered approach to ensure broad dissemination through national and local prehospital organisations and representative bodies. Furthermore, all the prehospital services have been approached to nominate a site champion who will ensure local dissemination of the survey. It may be difficult to reach smaller volunteer organisations and clinicians; however, the authors will use social media channels of national prehospital organisations to ensure maximum reach.

Potential sources of bias include self-selection bias, under-coverage of clinicians in services with lower survey reach, social desirability bias, and questionnaire interpretation bias. These risks will be mitigated by broad international dissemination, clear inclusion criteria, anonymous data collection, neutral wording, piloting of the survey by the core team, and transparent reporting of response patterns. Potential confounding will be addressed through multivariable analyses, with adjustment for relevant participant and service characteristics as described in the modelling strategy.

## Ethics

This study has received ethical approval to be conducted in Australia and New Zealand through the Southern Adelaide Local Health Network Human Research Ethics Committee (SALHN HREC) [2025/HRE00367], in accordance with the National Statement on Ethical Conduct in Human Research (2023) and the SA Health Research Ethics Policy. In addition, Health Research Authority (HRA) UK approval has been granted for participating UK sites [IRAS: 346781]. As of the date of submission, ethical approval is current and no amendments affecting approval status are pending.

## Dissemination

We anticipate that the primary findings of this study will be submitted for publication in a peer-reviewed emergency medicine or prehospital care journal. Findings will also be disseminated at national and international conferences relevant to prehospital care, resuscitation science, and emergency medicine. The study results will be shared with participating services and site champions to enable translation into local practice and policy where appropriate.

## Authors’ contributions

SN led study conceptualisation, overall study design, methodological development, data collection, coordination, and drafting of the protocol manuscript. SAQ contributed significantly across all stages, with involvement in study design, methodological refinement, protocol development, and critical revision of the manuscript. AB, NW, and AK contributed to protocol development, survey design, study implementation, and data acquisition. CG, DB, and LR contributed to study design, methodological refinement, data collection, and contributions to manuscript preparation. LA and TH contributed to protocol development, refinement of study materials, and critical revision of the manuscript. SU assisted in the formulation of the statistical analysis plan and determination of the sample size calculation strategy. MDC provided senior methodological oversight and ensured consistency across all stages. All authors contributed to the iterative critical revision of the manuscript and approved the final version for submission.

## Funding statement

This study has not received dedicated external grant funding from any public, commercial, or not-for-profit funding agency. The Chief Investigator, SN, has been allocated 0.1 FTE by the Emergency Department at Flinders Medical Centre to support project activities including protocol development, ethics submission, survey conduct, data analysis, and manuscript preparation. All other investigators are contributing their time voluntarily. SAQ has been allocated 0.1 FTE to support HREC approval, site coordination, data collection and analysis, and manuscript write-up. Costs associated with REDCap data management will be met through the Chief Investigator’s affiliation with Flinders University, which provides a REDCap subscription for this purpose. In addition, grant funding has been applied for to support the study activities; however, no funder has had, or will have, any role in study design, data collection, data analysis or interpretation, or in the writing or submission of publications arising from this study.

## CRediT authorship contribution statement

**Salman Naeem:** Writing – review & editing, Writing – original draft, Project administration, Methodology, Investigation, Formal analysis, Data curation, Conceptualization. **Stewart Ashcroft-Quinn:** Writing – review & editing, Writing – original draft, Methodology, Investigation, Formal analysis, Data curation, Conceptualization. **Alison Brown:** Writing – review & editing, Writing – original draft, Project administration, Methodology, Data curation, Conceptualization. **Amy Keir:** Writing – review & editing, Writing – original draft, Project administration, Methodology, Data curation, Conceptualization. **Nathan West:** Writing – review & editing, Writing – original draft, Methodology, Conceptualization. **Charmaine Gray:** Writing – review & editing, Writing – original draft, Resources, Project administration, Methodology, Investigation, Conceptualization. **Lisa Ramage:** Writing – review & editing, Writing – original draft, Resources, Project administration, Methodology, Data curation, Conceptualization. **Danyal Bakht:** Writing – review & editing, Writing – original draft, Methodology, Formal analysis, Data curation. **Leah Armit:** Writing – review & editing, Writing – original draft, Software, Resources, Methodology, Data curation, Conceptualization. **Tim Harris:** Writing – review & editing, Writing – original draft, Methodology, Conceptualization. **Shahid Ullah:** Writing – review & editing, Writing – original draft, Methodology, Investigation, Conceptualization. **Michael D. Christian:** Writing – review & editing, Writing – original draft, Supervision, Methodology, Conceptualization.

## Declaration of competing interest

The authors declare that they have no known competing financial interests, commercial relationships, or personal relationships that could have appeared to influence the design, conduct, or reporting of the work described in this protocol.

No author has received, or expects to receive, payment, honoraria, or other financial benefit from any organisation with a financial interest in the subject matter of this research.

## Appendix A Supplementary material

Supplementary File 1 PRECEPT-OHCA survey questionnaire assessing prehospital clinicians’ practices and confidence in identifying and managing reversible causes of out-of-hospital cardiac arrest.
