# Supplementary material for: PRECEPT-OHCA: prehospital evaluation of confidence and practice in the treatment of out-of-hospital cardiac arrest across Australia, the United Kingdom, and New Zealand: a cross-sectional study protocol
Source: Resusc Plus. 2026 Aug 8;31:101444. doi: 10.1016/j.resplu.2026.101444 (PMC13542911; doi:10.1016/j.resplu.2026.101444)
Supplement: Supplementary file 1 [file mmc1.pdf]

Prehospital evaluation of confidence and practice in treatment of out of hospital cardiac arrest across Australia, UK and New Zealand PRECEPT-OHCA

Participant information

Short Name of Research Project:
PRECEPT-OHCA

Full Name of Research Project:
Prehospital Evaluation of Confidence and Practice in Treatment of Out-of-Hospital Cardiac Arrest across Australia, UK and New Zealand

Sponsor Reference:

HREC in Australia: 2025/HRE00367

REC in UK: 26/HRA/1571

IRAS Project ID: 346781

V1.0 13.04.2026

Principal Investigator:
Dr Salman Naeem MBBS, FRCEM, FACEM, Dip IMC, PG Dip MUS

Chief Investigator UK

Mrs Alison Brown

Co-investigators

Dr Lisa Ramage

Professor Tim Harris

Dr Charmaine Gray

Dr Stewart Ashcroft Quinn

Ms Leah Armit

Professor Michael Christian

Project Sponsor:
Investigator initiated

Site Name:
All participating ambulance and aeromedical retrieval services across Australia, UK and New Zealand

---
What am I being invited to do?

You are invited to take part in a research project exploring clinician confidence, training, and practice in managing out-of-hospital cardiac arrest (OHCA). You have been invited because you are a clinician PHYSICALLY involved in prehospital care.

If you are not PHYSICALLY involved in OHCA management i.e. work in control-room (dispatch) only please ignore this survey.

If you take part, you will complete an anonymous online survey about your experience, training, and clinical decision-making in OHCA.

Around several hundred clinicians from multiple sites will take part across Australia, the United Kingdom and New Zealand.

Please read this information and ask us any questions. You can also talk to someone you trust, like a family member, friend, or your doctor. You can take some time to make up your mind. You get to decide whether this project is right for you.

Participation is voluntary.

What is the purpose of this project?

The purpose of this project is to understand how prehospital clinicians across Australia, the UK and New Zealand currently manage out-of-hospital cardiac arrest, including the makeup of response teams, the use of clinical adjuncts, and the availability of standard operating procedures. It also aims to assess clinicians' confidence in identifying the reversible causes of cardiac arrest (the 4 Hs and 4 Ts) and to identify the barriers they perceive in delivering optimal OHCA care. Ultimately, this study is part of bigger project aiming to identify gaps in current practice of OHCA management and then researching ways to improve the outcomes. This study seeks to highlight variation in practice, inform future training and guideline development, and identify opportunities to improve prehospital cardiac arrest outcomes.

Do I have to take part?

Participation is voluntary. You may stop at any time. Submission of the completed survey will
be considered implied consent. Because the survey is anonymous, responses cannot be withdrawn after submission.

What do I have to do?

You will complete a single online survey. The survey will take approximately 5-7 minutes to complete.
There are no restrictions or precautions you need to take for participation in this study.

Your time and expenses

Participation in this survey will be completely voluntary and will take about 5-7 minutes of your precious time. We really appreciate your time, however, you will not be compensated for participation in this survey.

Benefits

You may not directly benefit, but your responses will help improve understanding and training in OHCA management.

Risks

The survey poses minimal risk. Some questions may be sensitive. You may stop at any time.

Use of Information

Collecting your information

Anonymous information will be collected using the survey on REDCap database hosted by Flinders University. The information will be downloaded off the servers at the end of the study. It will be stored in password encrypted files on servers of Southern Adelaide Local Health Network (SALHN). The core team for the study will have access to it who will analyse the data and publish the results in peer-reviewed journal.

Keeping your information safe

To keep your information safe, we will:

follow all relevant privacy requirements

store information securely at SALHN servers with password encryption.

take steps to prevent anyone from accessing information that identifies you unless they are authorised to do so, such as the project team

We will keep your information for 15 years.

After this, we will destroy the information

Publishing project information

We will share certain information from this project so that others can use it and understand the study findings. This project information does not identify you individually. We will make this project information available through journal articles and presentations, By being in this project, you agree to us sharing project information for these purposes.

Future Data Sharing

No identifiable information will be shared. Anonymous data may be reused for future research.

Project Governance

This project is conducted by Dr Salman Naeem at Flinders Medical Centre and Flinders University. It has been approved by Southern Adelaide Local Health Network Hospital Research Ethics Committee and Health Research Authority UK.

Comments or Complaints

Contact: Dr Salman Naeem
Email: ohca2025@gmail.com

Do you consent to participate in the survey ?

◯ Yes
◯ No

Survey Questionnaire guidance

1. Answer each question honestly

There are no right or wrong answers. We are interested in your actual clinical practice, confidence level, and experiences.

2. Follow the instructions for each question type

"Mark only one option"
Select the single response that best describes you or your practice.

"Check all that apply"
You may choose multiple responses if applicable.

Free-text questions
Please write brief comments or explanations in the text box provided. Free text responses should not contain names, initials, patient details, or any information that could identify you or others

3. Base your answers on your usual clinical practice

If your role varies, answer according to how you most commonly work.

4. Skip questions only if they do not apply

Some questions depend on earlier responses (e.g., cardiac arrest exposure).
If a question does not apply to you, select the option that best fits (e.g., "None of the above") or leave the free-text box blank.

5. Be as accurate as possible

For frequency-based questions (daily/weekly/monthly etc.), choose the option closest to your typical practice.

6. Maintain confidentiality

Do not enter any patient-identifiable information.
The survey is anonymous, and no personal identifiers are collected.

Demographics

Please select your professional workgroup	○ Ambulance service personnel/ Paramedic<br>○ Nurse<br>○ Doctor

Please select the grade that you work at	◯ Community emergency responder / Community first responder<br>◯ Volunteer Prehospital Personnel/ Ambulance Community Officer<br>◯ Special Operations / Rescue / Flight Paramedic<br>◯ Paramedic<br>◯ Critical care paramedic/ Intensive care paramedic<br>◯ Other Ambulance Officer<br>◯ Registered Nurse<br>◯ Flight / Retrieval Nurse<br>◯ Medical retrieval registrar / Prehospital registrar / prehospital fellow<br>◯ Medical retrieval consultant / Prehospital consultant
Which service do you work for majority of your time	☐ Ambulance service<br>☐ Helicopter emergency medical service<br>☐ Prehospital retrieval service<br>☐ Volunteer emergency medical services (e.g. Mountain rescue, BASICS)
In which country do you practice ?	◯ Australia<br>◯ New Zealand<br>◯ England (United Kingdom)<br>◯ Wales (United Kingdom)<br>◯ Scotland (United Kingdom)<br>◯ Northern Ireland (United Kingdom)
How long have you worked as a prehospital clinician?	◯ < 5 years<br>◯ 5 - 9 years<br>◯ 10 - 14 years<br>◯ 15 - 19 years<br>◯ >20 years
Do you treat cardiac arrest as part of your usual practice ?	◯ Yes<br>◯ No
If yes, how often do you manage medical out-of-hospital cardiac arrest in your practice ?	◯ Daily<br>◯ Weekly<br>◯ Fortnightly<br>◯ Monthly<br>◯ Quarterly<br>◯ Bi-annually<br>◯ Annually<br>◯ Less than once per year
How often do you receive training (drills/simulation/workshop) for management of out-of-hospital cardiac arrest by your organisation?	◯ Daily<br>◯ Weekly<br>◯ Fortnightly<br>◯ Monthly<br>◯ Quarterly<br>◯ Bi-annually<br>◯ Annually<br>◯ Less than once per year<br>◯ Never

What is your highest level of resuscitation qualification ?

◯ Advanced Life Support -1 (ALS-1)
◯ Advanced Life Support-2 (ALS-2)
◯ Advanced Life Support (ALS)
◯ Basic Life Support (BLS)
◯ Immediate Life Support (ILS)
◯ Advanced Cardiac Life Support (ACLS)
◯ None

How many responders are typically part of the team for cardiac arrest ?

◯ 1
◯ 2
◯ 3
◯ 4
◯ 5
◯ >5

What equipment are you provided by your service for management of out-of-hospital cardiac arrest ?

☐ Automated external defibirillator (AED)
☐ Manual defibrillator
☐ Mechanical cardiac compression device (e.g. CorPuls, LUCAS etc)
☐ Bag-valve mask (BVM)
☐ Mechanical ventilator
☐ Supraglottic airway device (e,g. LMA, iGel)
☐ Endotracheal tube
☐ Basic airway equipment i.e. oropharyngeal (Geudel) and nasopharyngeal (NPA) airways
☐ Pocket mask
☐ Oxygen
☐ Point-of-care blood tests
☐ Point-of-care ultrasound
☐ Invasive blood pressure monitoring i.e. arterial line
☐ End-tidal CO2 monitoring
☐ Intraosseous access device
☐ Peripheral venous access
☐ Central venous access
☐ Front of neck access kit
☐ Thermometer (tympanic)
☐ Thermometer (rectal)
☐ None of the above

Which medications are you provided with by your service for management of out-of-hospital cardiac arrest?

☐ Adrenaline / Epinephrine
☐ Alteplase
☐ Amiodarone
☐ Calcium gluconate/ chloride
☐ Dextrose
☐ Esmolol
☐ Hypertonic saline
☐ Lidocaine / lignocaine
☐ Magnesium
☐ Normal saline/ hartmans fluid/ plasmalyte
☐ Potassium chloride
☐ Sodium bicarbonate
☐ Vasopressin
☐ Metaraminol
☐ Nor-adrenaline
☐ None

Confidence

Please rate your confidence from least confident to very confident on the 5-point Likert scale, in ruling in/out the 4Hs and 4Ts while managing out-of-hospital cardiac arrest in your current practice. By confidence we are trying to assess that using the resources in your team, how likely (confidently) are you able to rule in or out the 4Hs and 4Ts in OHCA management

How confident do you feel in managing out-of-hospital cardiac arrest?

- ◯ Not confident at all
- ◯ Less confident
- ◯ Unsure
- ◯ Somewhat confident
- ◯ Very confident

How do you rule in / out hypoxia as cause of cardiac arrest ?

____

How confident are you in ruling in Hypoxia as cause of OHCA?

- ◯ Not confident at all
- ◯ Less confident
- ◯ Unsure
- ◯ Somewhat confident
- ◯ Very confident

How confident are you in ruling out Hypoxia as cause of OHCA?

- ◯ Not confident at all
- ◯ Less confident
- ◯ Unsure
- ◯ Somewhat confident
- ◯ Very confident

How do you rule in / out hypovolemia as cause of cardiac arrest?

____

How confident are you in ruling in Hypovolemia as cause of OHCA?

- ◯ Not confident at all
- ◯ Less confident
- ◯ Unsure
- ◯ Somewhat confident
- ◯ Very confident

How confident are you in ruling out Hypovolemia as cause of OHCA?

- ◯ Not confident at all
- ◯ Less confident
- ◯ Unsure
- ◯ Somewhat confident
- ◯ Very confident

How do you rule in / out hyperkalemia as cause of cardiac arrest?

____

How confident are you in ruling in Hyperkalemia as cause of OHCA?

- ◯ Not confident at all
- ◯ Less confident
- ◯ Unsure
- ◯ Somewhat confident
- ◯ Very confident

How confident are you in ruling out Hyperkalemia as cause of OHCA?

- ◯ Not confident at all
- ◯ Less confident
- ◯ Unsure
- ◯ Somewhat confident
- ◯ Very confident

How do you rule in / out hypothermia as cause of cardiac arrest?

____

How confident are you in ruling in Hypothermia as cause of OHCA?

- ◯ Not confident at all
- ◯ Less confident
- ◯ Unsure
- ◯ Somewhat confident
- ◯ Very confident

How confident are you in ruling out Hypothermia as cause of OHCA?

- ◯ Not confident at all
- ◯ Less confident
- ◯ Unsure
- ◯ Somewhat confident
- ◯ Very confident

How do you rule in / out tension pneumothorax as cause of cardiac arrest?

____

How confident are you in ruling in Tension pneumothorax as cause of OHCA?

- ◯ Not confident at all
- ◯ Less confident
- ◯ Unsure
- ◯ Somewhat confident
- ◯ Very confident

How confident are you in ruling out Tension pneumothorax as cause of OHCA?

- ◯ Not confident at all
- ◯ Less confident
- ◯ Unsure
- ◯ Somewhat confident
- ◯ Very confident

How do you rule in / out cardiac tamponade as cause of cardiac arrest?

____

How confident are you in ruling in cardiac Tamponade as cause of OHCA?

- ◯ Not confident at all
- ◯ Less confident
- ◯ Unsure
- ◯ Somewhat confident
- ◯ Very confident

How confident are you in ruling out cardiac Tamponade as cause of OHCA?

- ◯ Not confident at all
- ◯ Less confident
- ◯ Unsure
- ◯ Somewhat confident
- ◯ Very confident

How do you rule in / out Toxins as cause of cardiac arrest?

____

How confident are you in ruling in Toxins as cause of OHCA?	◯ Not confident at all<br>◯ Less confident<br>◯ Unsure<br>◯ Somewhat confident<br>◯ Very confident
How confident are you in ruling out Toxins as cause of OHCA?	◯ Not confident at all<br>◯ Less confident<br>◯ Unsure<br>◯ Somewhat confident<br>◯ Very confident
How do you rule in / out thrombosis as cause of cardiac arrest?	__________________________________
How confident are you in ruling in Thrombosis as cause of OHCA?	◯ Not confident at all<br>◯ Less confident<br>◯ Unsure<br>◯ Somewhat confident<br>◯ Very confident
How confident are you in ruling out Thrombosis as cause of OHCA?	◯ Not confident at all<br>◯ Less confident<br>◯ Unsure<br>◯ Somewhat confident<br>◯ Very confident
How frequently do you need to deviate from standard ALS/2 guidelines to optimise patient outcomes?	◯ Always ◯ Often ◯ Sometimes<br>◯ Rarely ◯ Never
In which scenarios do you deviate from the standard ALS/2 guidelines?	__________________________________

Barriers to improvement in management of Out of Hospital Cardiac Arrest (OHCA)

Please provide information about your thoughts on barriers to improvement in your management of out-of-hospital cardiac arrest in the next section

In your opinion, what are the barriers to improving your management of out-of-hospital cardiac arrest?

- ☐ OHCA is managed well and there is little room for improvement
- ☐ I am not comfortable establishing ALS/2/ACLS
- ☐ I am comfortable establishing ALS/2/ACLS but cannot work beyond this due to current skills / knowledge
- ☐ I am comfortable establishing ALS/2/ACLS but cannot work beyond this due to organisational procedure & guidelines
- ☐ ALS/2/ACLS algorithm is not fit for OHCA
- ☐ Lack of human resources to effectively manage cardiac arrest
- ☐ Lack of point of care blood test to effectively rule in/out 4Hs and 4Ts
- ☐ Lack of point of care ultrasound to effectively rule in/out 4Hs and 4Ts
- ☐ Lack of training in cardiac arrest management
- ☐ Lack of advanced critical care resource/team
- ☐ Lack of ability to prescribe and administer certain drugs

Please expand on your top three barriers in improvement of OHCA management if not stated above

____

What additional resources, equipment, technical skills or protocols would enhance your ability to manage OHCA more effectively? (please name them only)

____

Is there anything else you would like to share about your experiences managing OHCA?

____

How did you hear about this study?

____
