# Supplementary material for: PRECEPT-OHCA: prehospital evaluation of confidence and practice in the treatment of out-of-hospital cardiac arrest across Australia, the United Kingdom, and New Zealand: a cross-sectional study protocol
Source: Resusc Plus. 2026 Aug 8;31:101444. doi: 10.1016/j.resplu.2026.101444 (PMC13542911; doi:10.1016/j.resplu.2026.101444)
Supplement: Supplementary file 2 [file mmc2.docx]

**Research Study Protocol**

**PRECEPT-OHCA**

Prehospital Evaluation of Confidence and Practice in Treatment of Out-of-Hospital Cardiac Arrest across Australia, the United Kingdom and New Zealand

| **Protocol version** | Version 1.3 |
| --- | --- |
| **Protocol date** | 05 December 2025 |
| **Chief Investigator** | Dr Salman Naeem |
| **Sponsor / coordinating site** | Emergency Department, Flinders Medical Centre / Southern Adelaide Local Health Network |
| **Study design** | Multicentre, international, cross-sectional survey |
| **Study countries** | Australia, New Zealand and United Kingdom |

**OFFICIAL**

# Document control

| **Version** | **Date** | **Description** | **Author/owner** |
| --- | --- | --- | --- |
| 1.3 | 05/12/2025 | Professionalised protocol draft based on existing SALHN protocol template content. | Study team |

# Table of contents

Document control	2

Abbreviations	3

1. Protocol synopsis	3

2. Administrative information	3

3. Background and rationale	4

4. Research question, aims and objectives	5

5. Outcomes	6

6. Study design and setting	6

7. Study population and eligibility	7

8. Recruitment and participant activities	7

9. Consent	7

10. Data collection and variables	8

11. Sample size	9

12. Statistical analysis plan	9

13. Data management, privacy and confidentiality	10

14. Ethics and governance considerations	11

15. Timeline and project end point	11

16. Reporting, dissemination and future plans	11

17. References	11

Appendix 1. Planned participating services	13

# Abbreviations

| **Abbreviation** | **Definition** |
| --- | --- |
| **ALS** | Advanced life support |
| **AUS-ROC** | Australasian Resuscitation Outcomes Consortium |
| **CI** | Chief Investigator |
| **CPR** | Cardiopulmonary resuscitation |
| **EMS** | Emergency medical services |
| **eCPR** | Extracorporeal cardiopulmonary resuscitation |
| **GCP** | Good Clinical Practice |
| **HREC** | Human Research Ethics Committee |
| **ILCOR** | International Liaison Committee on Resuscitation |
| **OHCA** | Out-of-hospital cardiac arrest |
| **PoCUS** | Point-of-care ultrasound |
| **REDCap** | Research Electronic Data Capture |

# 1. Protocol synopsis

| **Project title** | PRECEPT-OHCA: Prehospital Evaluation of Confidence and Practice in Treatment of Out-of-Hospital Cardiac Arrest across Australia, the United Kingdom and New Zealand |
| --- | --- |
| **Study design** | Multicentre, international, observational, cross-sectional survey of prehospital clinicians. |
| **Setting** | Ambulance, retrieval, air ambulance and volunteer prehospital services across Australia, New Zealand and the United Kingdom. |
| **Target population** | Registered or accredited prehospital clinicians who physically attend and deliver clinical care at the scene of an out-of-hospital cardiac arrest. |
| **Primary outcome** | Perceived confidence in ruling in and ruling out each of the 4Hs and 4Ts, measured using five-point Likert-scale items. |
| **Secondary outcomes** | Service-level OHCA standard operating procedures; availability and use of adjuncts such as PoCUS, point-of-care blood tests and mechanical CPR devices; and perceived barriers to improving OHCA management. |
| **Recruitment approach** | Participating organisations will distribute an anonymous REDCap survey through site champions, email invitations, posters with QR codes, and approved social media promotion. |
| **Minimum sample size** | A minimum of 382 completed responses is required based on a finite population estimate of 54,874 clinicians, 95% confidence level, p=0.5 and 5% margin of error. |
| **Estimated duration** | Survey open from 1 July 2026 to 31 August 2026. |
| **Data handling** | Anonymous survey data will be collected in REDCap, hosted through Flinders University, and analysed by authorised study investigators. |

# 2. Administrative information

## 2.1 Chief Investigator and coordinating responsibilities

The Chief Investigator, Dr Salman Naeem, will be responsible for overall study design, coordination, ethics and governance submissions, survey conduct, data collection oversight, data analysis, manuscript preparation and dissemination. The Chief Investigator has 0.1 FTE allocated through the Emergency Department at Flinders Medical Centre to support this project and will supervise a medical student allocated through Flinders University to assist with the study.

## 2.2 Project team and roles

| **Name** | **Affiliation / site** | **Role** | **Study responsibilities** |
| --- | --- | --- | --- |
| Dr Salman Naeem | Emergency Department, Flinders Medical Centre | Chief Investigator | Overall study oversight, ethics and governance, survey conduct, analysis, manuscript preparation and publication. |
| Dr Nathan West | MedSTAR Emergency Medical Retrieval Service | Site Principal Investigator | MedSTAR site oversight and survey dissemination among eligible clinicians. |
| Associate Professor Amy Keir | South Australian Ambulance Service | Site Principal Investigator | SAAS site oversight and survey dissemination among eligible clinicians. |
| Professor Tim Harris | Central Adelaide Local Health Network / MedSTAR Retrieval Consultant | Associate Investigator | Core study team member; support study execution, interpretation and manuscript development. |
| Dr Charmaine Gray | Southern Adelaide Local Health Network | Associate Investigator | Support HREC approvals, coordination, data analysis and manuscript preparation. |
| Ms Leah Armit | Southern Adelaide Local Health Network | Research Nurse Coordinator / Associate Investigator | Support HREC approval, site coordination, data collection, analysis and write-up. |
| Dr Stewart Ashcroft Quinn | Southern Adelaide Local Health Network | Associate Investigator | Support HREC approval, site coordination, data collection, analysis and manuscript preparation. |

All listed investigators will maintain Good Clinical Practice certification as required by the National Clinical Trials Governance Framework. Roles and site-specific responsibilities will be documented in the study delegation log.

## 2.3 Resources and funding

The study will be supported through in-kind investigator time. The Chief Investigator has allocated research time within his employment plan. Other investigators will contribute time in-kind. No external commercial funding is currently provided. Any future funding will be declared and will not influence study design, data analysis, interpretation, manuscript writing or dissemination of results.

# 3. Background and rationale

## 3.1 Clinical background

Out-of-hospital cardiac arrest is a time-critical emergency associated with substantial morbidity and mortality. OHCA is defined as cardiac arrest occurring outside hospital and is confirmed clinically by the absence of signs of circulation. International registry data show that survival remains poor despite advances in resuscitation systems, prehospital capability and post-arrest care.

The International Liaison Committee on Resuscitation and Resuscitation Council UK recommend consideration and treatment of potentially reversible causes of cardiac arrest, commonly framed as the 4Hs and 4Ts: hypoxia, hypovolaemia, hypo- or hyperkalaemia and other metabolic disturbances, hypothermia, tension pneumothorax, cardiac tamponade, toxins and thrombosis. These recommendations are embedded in advanced life support algorithms, but diagnostic certainty is often difficult to achieve in the prehospital environment.

## 3.2 Literature context

Prehospital resuscitation systems have changed significantly over recent decades. Mechanical chest compression devices, advanced ventilators, point-of-care diagnostics, point-of-care ultrasound, critical care paramedics, prehospital physicians and extracorporeal cardiopulmonary resuscitation pathways have expanded the capabilities of some services. However, adoption is variable across regions and organisations. The composition of OHCA response teams and the availability of diagnostic adjuncts may influence clinicians’ ability to identify and treat reversible causes during resuscitation.

Existing OHCA registries commonly record presumed aetiology, but these data are often incomplete, inconsistently reported or biased toward patients who survive long enough to receive in-hospital investigations. There is therefore limited direct evidence about how prehospital clinicians identify, rule in or rule out reversible causes during OHCA resuscitation, or how confident they are in doing so.

The perspectives of prehospital clinicians are central to understanding real-world barriers to improving OHCA care. Challenges may include limited access to history, diagnostic uncertainty, time pressure, scene constraints, resource availability, variable training exposure and differences in team composition. A structured international survey can describe current practice, identify service variation, and generate hypotheses for future interventional or implementation research.

## 3.3 Study rationale

No previous study has comprehensively evaluated prehospital clinicians’ confidence in identifying the underlying cause of OHCA across Australia, New Zealand and the United Kingdom. This study will describe the current landscape of OHCA management, including response team composition, clinician experience, use of diagnostics and adjuncts, confidence in identifying reversible causes, and perceived barriers to improving outcomes. The results will support targeted education, protocol development, service benchmarking and future research into diagnostic and treatment strategies for OHCA.

# 4. Research question, aims and objectives

## 4.1 Research questions

The PRECEPT-OHCA study will address three principal research questions:

1. What is the current composition of prehospital teams responding to OHCA across ambulance and retrieval services in Australia, the United Kingdom and New Zealand, including the use of clinical adjuncts and service-level standard operating procedures?
2. What is prehospital clinicians' perceived confidence in ruling in or ruling out each reversible cause of OHCA, commonly referred to as the 4Hs and 4Ts (hypoxia, hypovolaemia, hypo-/hyperkalaemia, hypothermia, tension pneumothorax, tamponade, thrombosis and toxins)?
3. What are prehospital clinicians' perceived barriers to improving outcomes in OHCA management?

## 4.2 Aim

1. To assess clinician confidence in identifying reversible causes of OHCA and to describe perceived barriers to improving OHCA management.
2. To describe the current composition of prehospital teams responding to OHCA across participating services in Australia, New Zealand and the United Kingdom.

## 4.3 Objectives

1. Describe clinician confidence in ruling in and ruling out each of the 4Hs and 4Ts during OHCA management.
2. Compare confidence across prespecified clinician and service-level groups, including professional role, years of experience, service type, frequency of OHCA exposure, training frequency, country and availability or use of PoCUS.
3. Describe team composition, standard operating procedures and availability of adjuncts used in OHCA management across participating services.
4. Identify perceived barriers to improving OHCA management using structured survey items and free-text responses.
5. Generate evidence to inform future training, protocol standardisation, service development and interventional research aimed at improving OHCA outcomes.

# 5. Outcomes

## 5.1 Primary outcome

The primary outcome is the perceived confidence of prehospital clinicians in ruling in and ruling out each reversible cause of OHCA: the 4Hs (hypoxia, hypovolaemia, hypo-/hyperkalaemia and hypothermia) and the 4Ts (tension pneumothorax, tamponade, thrombosis and toxins). Confidence will be measured using five-point Likert-scale survey items.

## 5.2 Secondary outcomes

1. Description of current service-level standard operating procedures or protocols for OHCA management across participating services.
2. Prevalence and patterns of use of clinical adjuncts, including PoCUS, point-of-care blood investigations and mechanical CPR devices.
3. Prehospital clinicians' perceived barriers to improving outcomes in OHCA management.

# 6. Study design and setting

## 6.1 Design

PRECEPT-OHCA is a multicentre, international, observational, cross-sectional survey study of prehospital clinicians. It is non-interventional and will collect anonymous, self-reported data through an online REDCap survey. The survey will be accessible for responses from 1 July 2026 to 31 August 2026.

## 6.2 Setting

The study will be conducted across ambulance, air ambulance, retrieval and volunteer medical services in Australia, New Zealand and the United Kingdom. Coordination across Australia and New Zealand will be facilitated through the Australasian College of Paramedicine (ACP) and the Australasian College for Emergency Medicine (ACEM). United Kingdom sites will be engaged through the National HEMS Research and Audit Forum (NHRAF) and the National Ambulance Research Steering Group UK (NARSG UK).

## 6.3 Participating services

The study will aim to include major primary prehospital response organisations and retrieval/air ambulance services across the three participating regions. The planned service list is provided in Appendix 1. Final participating organisations will depend on organisational approvals, site champion engagement and local governance requirements.

# 7. Study population and eligibility

## 7.1 Study population

For this study, a prehospital clinician is defined as a registered or accredited healthcare professional, including a paramedic, emergency medical technician, retrieval physician or retrieval nurse, who physically attends and delivers clinical care at the scene of an OHCA, whether employed by a statutory ambulance service, an air ambulance or retrieval service, or a volunteer medical service.

## 7.2 Inclusion criteria

- All prehospital clinicians physically involved in the management of OHCA.

## 7.3 Exclusion criteria

- Prehospital clinicians not physically involved in the management of OHCA.

# 8. Recruitment and participant activities

## 8.1 Identification of potential participants

- Potential participants will self-identify as eligible based on the inclusion and exclusion criteria displayed in the survey invitation, participant information material and recruitment poster. No patient data will be accessed and no participant pre-screening will occur before ethics and governance authorisation.

## 8.2 Recruitment strategy

- Prehospital organisations and clinical or research leads will be invited through ACP, ACEM, NHRAF and NARSG UK and other approved national prehospital and emergency medicine networks.
- Each participating organisation will identify a site champion or local lead to support survey dissemination.
- Site champions will distribute the REDCap survey using email links and QR codes embedded in approved departmental posters displayed at service locations.
- The survey will also be advertised through approved professional and social media channels. Using multiple official communication routes and inviting clinicians across participating organisations will help mitigate sampling and under-coverage bias.
- Participation will be voluntary, anonymous and uncompensated.

## 8.3 Participant commitment

Participants will complete a single anonymous online survey. No study visits, clinical procedures, tissue samples, device use or follow-up will be required. No reimbursement will be provided for time, travel or parking.

## 8.4 Co-enrolment

Participants may participate in other studies or service evaluations because this study is a cross-sectional anonymous survey and does not involve clinical interventions.

# 9. Consent

Given the fully anonymised nature of the study, formal written consent will not be required. Participant information, eligibility criteria and consent information will be displayed digitally at the beginning of the REDCap survey. Participation will be voluntary, and completion and submission of the survey will constitute implied consent. The study team is not requesting a waiver of consent.

Because the survey is anonymous and no identifying information will be collected, participants will not be able to withdraw their submitted data after survey completion. This limitation will be clearly explained in the participant information statement before consent.

# 10. Data collection and variables

## 10.1 Survey platform

A structured, anonymised, self-administered electronic questionnaire will be hosted on REDCap through a secure Flinders University account accessible only to authorised study investigators. Participants will access the survey using a hyperlink or QR code. REDCap will be configured so responses cannot be linked to individual participants, and the survey will not collect names, contact details or other direct identifiers.

## 10.2 Survey development and pilot assessment

The survey was developed by the core research team to address the primary and secondary outcomes, using the 4Hs and 4Ts framework as the structural basis for the confidence-related items. Item generation was informed by a review of the existing literature and by the team's clinical and methodological expertise in prehospital and emergency care. Draft items were iteratively reviewed and refined by the co-investigator group. Before full distribution, the core research team pilot-tested the questionnaire to assess clarity, face validity and neutrality of wording.

## 10.3 Survey domains

- Participant professional role, years of experience, country and service type.
- Frequency of exposure to OHCA and frequency of OHCA-specific training.
- Team composition and available clinical roles during OHCA response.
- Availability and use of adjuncts, including PoCUS, point-of-care blood testing, mechanical CPR and other diagnostic or treatment supports.
- Confidence in ruling in and ruling out each reversible cause of OHCA using five-point Likert-scale items.
- Methods used by clinicians to identify reversible causes.
- Perceived barriers to improving OHCA management, including free-text responses.
- Service-level OHCA standard operating procedures or protocols, where available.

## 10.4 Service protocol collection

Prehospital services across the three countries will be invited to share their OHCA standard operating procedure or management protocol where local policy permits. These documents will contextualise clinician-reported responses and describe service-level variation in OHCA management pathways and the availability of diagnostic or treatment adjuncts.

## 10.5 Data quality and preparation

After survey closure, data will be exported from REDCap and checked for completeness, consistency and plausibility before datasets are amalgamated and analysed. The final analysis dataset will be accessible only to authorised members of the study team.

# 11. Sample size

The estimated total survey population across the three countries is 54,874 individuals, comprising approximately 26,071 ambulance service staff in the United Kingdom, 26,603 paramedics in Australia and 2,200 paramedics in New Zealand. Because no previous large international survey has quantified clinician confidence in this context, a conservative prevalence estimate of 50% will be assumed to maximise the required sample. Using a two-sided 95% confidence level (Z = 1.96), p = 0.5 and a 5% margin of error gives an initial uncorrected sample size of n₀ = 384. Applying finite population correction for 54,874 eligible clinicians yields a minimum required sample of 382 completed responses. This will estimate the proportion reporting confidence with acceptable precision. Composite confidence scores will also be analysed, but no separate sample-size calculation will be performed because evidence is insufficient regarding their expected distribution, variability or clinically meaningful differences. Composite-score analyses will therefore be supportive of the primary outcome.

# 12. Statistical analysis plan

## 12.1 General principles

Analyses will be conducted using Microsoft Excel and IBM SPSS Statistics version 27 or later. Statistical tests will be two-sided, with p < 0.05 considered statistically significant. Interpretation will prioritise effect sizes, 95% confidence intervals, clinical relevance and consistency across analyses rather than p-values alone. Distributions of continuous variables will be assessed using histograms, Q-Q plots and appropriate statistical tests before analysis.

## 12.2 Descriptive analysis

Normally distributed continuous variables will be summarised using means and standard deviations (SD). Non-normally distributed continuous variables and ordinal variables will be summarised using medians and interquartile ranges (IQR). Categorical variables will be reported using frequencies and percentages.

## 12.3 Primary outcome analysis

Confidence will be analysed for each individual reversible cause using the five-point Likert-scale responses, composite confidence scores and a dichotomised confidence outcome. Composite rule-in and rule-out confidence scores will be derived from the corresponding Likert-scale items, with higher scores indicating greater overall confidence. Individual ratings for each 4H and 4T will also be summarised descriptively. Internal consistency will be considered acceptable when Cronbach's alpha is at least 0.70.

## 12.4 Group comparisons

Confidence outcomes will be compared across prespecified groups: years of prehospital experience, professional group, service type, frequency of OHCA exposure, organisational training frequency, country, and availability or use of PoCUS. Composite confidence scores will be summarised using measures appropriate to their distribution. For two-group comparisons, Student's t-test will be used for normally distributed continuous data and the Mann-Whitney U test for non-normally distributed data. Comparisons across more than two groups will use the Kruskal-Wallis test, with post-hoc pairwise comparisons adjusted for multiple testing where appropriate.

For the dichotomised analysis, high confidence will comprise responses of 'somewhat confident' or 'very confident'; lower confidence will comprise 'unsure', 'less confident' or 'not confident at all'. Dichotomised confidence outcomes will be compared using Pearson's chi-squared test or Fisher's exact test, as appropriate. Results will be reported with effect estimates, 95% confidence intervals and p-values.

## 12.5 Regression modelling

Regression modelling will examine factors independently associated with clinician confidence. Composite confidence scores will be analysed using multivariable linear regression if model assumptions are adequately met; model fit will be assessed using residual diagnostics and adjusted R². Dichotomised confidence outcomes will be analysed using mixed-effects logistic regression and reported as odds ratios with 95% confidence intervals. Goodness of fit will be assessed using the Hosmer-Lemeshow test and discrimination using the area under the receiver operating characteristic curve (AUC). Candidate explanatory variables will include years of prehospital experience, professional group, service type, frequency of OHCA exposure, organisational training frequency, country, and availability or use of PoCUS.

## 12.6 PoCUS subgroup analysis

A prespecified subgroup analysis will compare confidence outcomes between clinicians with and without access to PoCUS. It will examine confidence in ruling in and ruling out cardiac tamponade, hypovolaemia, tension pneumothorax and thrombosis, as these conditions may be directly informed by PoCUS. Analyses will adjust for experience, professional group, service type, training frequency, OHCA exposure frequency and country of practice, and will use the statistical methods specified above.

## 12.7 Qualitative analysis

Free-text responses will undergo inductive thematic analysis. AI-assisted coding tools may be used to facilitate data organisation, but the research team will review and refine all themes. Qualitative themes will be triangulated with quantitative findings to provide a comprehensive account of clinician perspectives and a richer interpretation of practice variation and perceived barriers.

## 12.8 Missing data

The extent and pattern of missing data will be described for each variable. Complete-case analysis will be used for the primary analyses. If missing data are substantial or appear systematically related to key participant or service characteristics, sensitivity analyses will assess the potential effect of missingness. Where appropriate, multiple imputation may be used as a sensitivity analysis to evaluate the robustness of findings to assumptions about missing data.

## 12.9 Bias and confounding

Potential sources of bias include self-selection bias, under-coverage of clinicians in services with lower survey reach, social desirability bias and questionnaire interpretation bias. These risks will be mitigated by broad international dissemination, clear inclusion criteria, anonymous data collection, neutral wording, piloting of the survey by the core team and transparent reporting of response patterns. Confounding will be addressed in prespecified adjusted analyses where feasible, particularly for comparisons involving PoCUS access and clinician confidence.

# 13. Data management, privacy and confidentiality

The study will collect anonymous survey data. No direct participant identifiers will be collected. REDCap access will be restricted to authorised investigators. Data will be exported for analysis in de-identified aggregate form and stored in accordance with Flinders University, SA Health and SALHN data governance requirements, including applicable requirements for secure storage, access control, retention and disposal of research records.

No data linkage is planned or anticipated. No biological samples, tissue samples or genetic testing are involved in this study.

# 14. Ethics and governance considerations

## 14.1 Risk profile

This is a low-risk anonymous survey of health professionals about clinical practice, confidence and perceived barriers. The study does not involve patients, patient records, clinical interventions or identifiable participant data.

## 14.2 Dependent or unequal relationships

No dependent or unequal relationship is expected between participants and researchers. Participation will be voluntary, anonymous and not monitored by employers or service leads.

## 14.3 Conflicts of interest

No conflicts of interest have been declared. Any future actual, potential or perceived conflicts will be declared to the relevant ethics and governance bodies and managed in accordance with institutional policy.

## 14.4 Consumer and community engagement

Consumer engagement has informed the design of this study. A consumer engagement meeting was held on 10 July 2025. Consumer representatives emphasised the importance of understanding how OHCA services are currently delivered and identifying barriers faced by clinicians in improving care. Consumers also supported the use of social media to promote the survey and increase its reach and impact.

# 15. Timeline and project end point

| **Ethics and governance approvals** | Estimated 6 months |
| --- | --- |
| **Survey open period** | 1 July 2026 to 31 August 2026 |
| **Analysis and manuscript preparation** | Estimated 3 months after survey closure |
| **End of project** | Defined as manuscript submission for publication |

# 16. Reporting, dissemination and future plans

Findings will be reported in aggregate form and disseminated through peer-reviewed publication, conference presentation and feedback to participating organisations and stakeholder networks. The results will identify practice variation, diagnostic confidence gaps and perceived barriers to OHCA management. These findings will support future work on targeted education, protocol standardisation, implementation strategies and interventional studies to improve OHCA care and outcomes.

The study team will comply with annual and final reporting obligations, amendment reporting and applicable requirements of the National Statement on Ethical Conduct in Human Research, SA Health research governance policy and institutional research integrity frameworks.

# 17. References

- Yan S, Gan Y, Jiang N, Wang R, Chen Y, Luo Z, et al. The global survival rate among adult out-of-hospital cardiac arrest patients who received cardiopulmonary resuscitation: a systematic review and meta-analysis. Critical Care. 2020;24(1).
- Bray J, Howell S, Ball S, Doan T, Bosley E, Smith K, et al. The epidemiology of out-of-hospital cardiac arrest in Australia and New Zealand: a binational report from the Australasian Resuscitation Outcomes Consortium (Aus-ROC). Resuscitation. 2022;172:74-83.
- Paratz ED, Smith K, Ball J, van Heusden A, Zentner D, Parsons S, et al. The economic impact of sudden cardiac arrest. Resuscitation. 2021;163:49-56.
- Berg KM, Bray JE, Ng KC, Liley HG, Greif R, Carlson JN, et al. 2023 International Consensus on Cardiopulmonary Resuscitation and Emergency Cardiovascular Care Science With Treatment Recommendations. Circulation. 2023;148(24):e187-e280.
- Andersen LW, Holmberg MJ, Berg KM, Donnino MW, Granfeldt A. In-hospital cardiac arrest. JAMA. 2019;321(12):1200-1210.
- Meir Tabi, Perel N, Taha L, Amsalem I, Hitter R, Maller T, et al. Out of hospital cardiac arrest - new insights and a call for a worldwide registry and guidelines. BMC Emergency Medicine. 2024;24(1).
- Hawkes C, Booth S, Ji C, Brace-McDonnell SJ, Whittington A, Mapstone J, et al. Epidemiology and outcomes from out-of-hospital cardiac arrests in England. Resuscitation. 2017;110:133-140.
- Jones A, Donald MJ, Jansen JO. Evaluation of the provision of helicopter emergency medical services in Europe. Emergency Medicine Journal. 2018;35(12):720-725.
- Poole K, Couper K, Smyth MA, Yeung J, Perkins GD. Mechanical CPR: Who? When? How? Critical Care. 2018;22(1).
- Yacoub S. The use of point-of-care ultrasound for out-of-hospital cardiac arrest management: a review of the literature. Current Emergency and Hospital Medicine Reports. 2024;12(4):125-131.
- Holmberg MJ, Granfeldt A, Guerguerian AM, Sandroni C, Hsu CH, Gardner RM, et al. Extracorporeal cardiopulmonary resuscitation for cardiac arrest: an updated systematic review. Resuscitation. 2023;182:109665.
- Regional variation in the characteristics, incidence and outcomes of out-of-hospital cardiac arrest in Australia and New Zealand: results from the Aus-ROC Epistry. Resuscitation. 2018;126:49-57.
- Schwaiger D, Krösbacher A, Eckhardt C, Schausberger L, Baubin M, Rajsic S. Out-of-hospital cardiac arrest: a 10-year analysis of survival and neurological outcomes. Heart & Lung. 2025;73:1-8.
- Tang L, Gu WJ, Wang F. Mechanical versus manual chest compressions for out-of-hospital cardiac arrest: a meta-analysis of randomized controlled trials. Scientific Reports. 2015;5(1).
- Oh YT, Lee CA, Park HA, Park J, Kim S, Park HJ, et al. Effectiveness of chest compression-synchronized ventilation in patients with cardiac arrest. Journal of Clinical Medicine. 2025;14(7):2394.
- Kruit N, Ferguson I, Dieleman J, Burns B, Shearer N, Tian D, et al. Use of transoesophageal echocardiography in the pre-hospital setting to determine compression position in out of hospital cardiac arrest. Resuscitation. 2025;209:110582.
- Boulton AJ, Edwards R, Gadie A, Clayton D, Leech C, Smyth MA, et al. Prehospital critical care beyond advanced life support for out-of-hospital cardiac arrest: a systematic review. Resuscitation Plus. 2024;21:100803.
- Wohlgemut JM, Marsden MER, Stoner RS, Pisirir E, Kyrimi E, Grier G, et al. Diagnostic accuracy of clinical examination to identify life- and limb-threatening injuries in trauma patients. Scandinavian Journal of Trauma, Resuscitation and Emergency Medicine. 2023;31(1).
- Missel AL, Dowker SR, Dzierwa D, Krein SL, Coulter-Thompson EI, Williams M, et al. Factors impacting treatment of out-of-hospital cardiac arrest: a qualitative study of emergency responders. Journal of the American Heart Association. 2023;12(10).
- Office for National Statistics. The healthcare workforce across the UK. 2024.
- Scottish Ambulance Service. Increase in numbers of newly qualified paramedics. 2025.
- Australian Health Practitioner Regulation Agency. Annual report 2025.
- Paramedic Council of New Zealand. Paramedic statistics. 2025.
- Hendra R, Hill A. Rethinking response rates: new evidence of little relationship between survey response rates and nonresponse bias. Evaluation Review. 2018;43(5):307-330.

# Appendix 1. Planned participating services

## Australia

- Australian Capital Territory Ambulance Service
- Ambulance Tasmania
- Ambulance Victoria
- New South Wales Ambulance
- Queensland Ambulance Service
- South Australian Ambulance Service
- St John Ambulance Australia (Northern Territory)
- Sydney Helicopter Emergency Medical Service
- MedSTAR Emergency Medical Retrieval Service

## New Zealand

- Auckland Helicopter Medical Service
- Hato Hone St John
- Wellington Free Ambulance

## United Kingdom - Ambulance services

- East Midlands Ambulance Service
- East of England Ambulance Service
- Great Western Ambulance Service
- London Ambulance Service
- North East Ambulance Service
- North West Ambulance Service
- South Central Ambulance Service
- South East Coast Ambulance Service
- South Western Ambulance Service
- Staffordshire Ambulance Service
- West Midlands Ambulance Service
- Yorkshire Ambulance Service
- Welsh Ambulance Service
- Scottish Ambulance Service

## United Kingdom - Air ambulance and retrieval services

- Cornwall Air Ambulance
- The Air Ambulance Service
- Devon Air Ambulance
- Dorset and Somerset Air Ambulance
- East Anglian Air Ambulance
- Essex & Herts Air Ambulance
- Great Western Air Ambulance
- Hampshire and Isle of Wight Air Ambulance
- Air Ambulance Kent Surrey Sussex
- Magpas Air Ambulance
- Lincolnshire & Nottinghamshire Air Ambulance
- Midlands Air Ambulance
- Scottish Air Ambulance
- Wales Air Ambulance
- Yorkshire Air Ambulance
- Wiltshire and Bath Air Ambulance Charity
- Thames Valley Air Ambulance
- Scotland's Charity Air Ambulance
- Northern Ireland Air Ambulance
- London's Air Ambulance
- Great North Air Ambulance
